# Adiponectin Attenuates Lipopolysaccharide-induced Apoptosis by Regulating the Cx43/PI3K/AKT Pathway

**DOI:** 10.3389/fphar.2021.644225

**Published:** 2021-05-18

**Authors:** Luqian Liu, Meijuan Yan, Rui Yang, Xuqing Qin, Ling Chen, Li Li, Junqiang Si, Xinzhi Li, Ketao Ma

**Affiliations:** ^1^ Key Laboratory of Xinjiang Endemic and Ethnic Diseases, Ministry of Education, Shihezi University School of Medicine, Shihezi, China; ^2^ NHC Key Laboratory of Prevention and Treatment of Central Asia High Incidence Diseases, First Affiliated Hospital, Shihezi University School of Medicine, Shihezi, China; ^3^ Department of Pathophysiology, Shihezi University School of Medicine, Shihezi, China; ^4^ Department of Physiology, Shihezi University School of Medicine, Shihezi, China

**Keywords:** adiponectin, apoptosis, sepsis, PI3K/AKT, connexin43

## Abstract

Cardiomyocyte apoptosis is a crucial factor leading to myocardial dysfunction. Adiponectin (APN) has a cardiomyocyte-protective impact. Studies have shown that the connexin43 (Cx43) and phosphatidylinositol-3-kinase (PI3K)/protein kinase B (AKT) signaling pathways play an important role in the heart, but whether APN plays a protective role by regulating these pathways is unclear. Our study aimed to confirm whether APN protects against lipopolysaccharide (LPS)-induced cardiomyocyte apoptosis and to explore whether it plays an important role through regulating the Cx43 and PI3K/AKT signaling pathways. In addition, our research aimed to explore the relationship between the Cx43 and PI3K/AKT signaling pathways. *In vitro* experiments: Before H9c2 cells were treated with LPS for 24 h, they were pre-treated with APN for 2 h. The cytotoxic effect of APN on H9c2 cells was evaluated by a CCK-8 assay. The protein levels of Bax, Bcl2, cleaved caspase-3, cleaved caspase-9, Cx43, PI3K, p-PI3K, AKT and p-AKT were evaluated by Western blot analysis, and the apoptosis rate was evaluated by flow cytometry. APN attenuated the cytotoxicity induced by LPS. LPS upregulated Bax, cleaved caspase-3 and cleaved caspase-9 and downregulated Bcl2 in H9c2 cells; however, these effects were attenuated by APN. In addition, LPS upregulated Cx43 expression, and APN downregulated Cx43 expression and activated the PI3K/AKT signaling pathway. LPS induced apoptosis and inhibited PI3K/AKT signaling pathway in H9c2 cells, and these effects were attenuated by Gap26 (a Cx43 inhibitor). Moreover, the preservation of APN expression was reversed by LY294002 (a PI3K/AKT signaling pathway inhibitor). *In vivo* experiments: In C57BL/6J mice, a sepsis model was established by intraperitoneal injection of LPS, and APN was injected into enterocoelia. The protein levels of Bax, Bcl2, cleaved caspase-3, and Cx43 were evaluated by Western blot analysis, and immunohistochemistry was used to detect Cx43 expression and localization in myocardial tissue. LPS upregulated Bax and cleaved caspase-3 and downregulated Bcl2 in sepsis; however, these effects were attenuated by APN. In addition, the expression of Cx43 was upregulated in septic myocardial tissue, and APN downregulated Cx43 expression in septic myocardial tissue. In conclusion, both *in vitro* and *in vivo*, the data demonstrated that APN can protect against LPS-induced apoptosis during sepsis by modifying the Cx43 and PI3K/AKT signaling pathways.

## Introduction

Numerous studies have shown that sepsis is a multiple organ dysfunction caused by the body’s immune response to microbial infections. Sepsis is an extremely rapid and deadly disease with high morbidity and mortality (Merx and Weber, 2007; Sammon et al., 2015). Sepsis is often accompanied by organ damage and organ failure. Sepsis-induced myocardial dysfunction (SIMD) is a common complication in patients with sepsis. Many studies have shown that approximately 50% of patients with sepsis show signs of myocardial insufficiency, and the repair of myocardial function affects the prognosis of patients with sepsis (Poveda-Jaramillo, 2021). Compared with patients without cardiovascular dysfunction, sepsis patients with myocardial dysfunction have an increased mortality rate (by 3-fold), and these patients usually show myocarditis, abnormal contractility, increased interstitial collagen and mitochondrial damage (Blanco et al., 2008; Alvarez et al., 2016). Moreover, due to the complexity of myocardial injury in sepsis, its pathophysiological mechanism is not yet fully understood. Lipopolysaccharide (LPS), a major endotoxin derived from Gram-negative bacteria, has been widely used to induce sepsis myocardial injury models *in vivo* and *in vitro* (Luo et al., 2020)**.** LPS induces cardiac dysfunction and the production of inflammatory factors (Fallach et al., 2010)**.**

Adiponectin (APN) is widely expressed in myocardial tissues and has cardioprotective effects *in vivo* and *in vitro* (Dong et al., 2012; Essick Eric et al., 2013; Ghantous et al., 2015) through its anti-inflammatory and anti-atherosclerotic effects. It can protect the heart by reducing inflammation and preventing damage caused by various mediators (Han & Judd Robert, 2018). A study showed that APN can reduce the secretion of TNF-α and apoptosis induced by LPS in rat cardiomyocytes (Shibata et al., 2005). In addition, APN inhibits ROS-induced cardiac remodeling of rat ventricular myocytes by activating AMPK and inhibiting ERK signalling and NF-kB activation (Essick et al., 2011). Connexin43 (Cx43)-mediated gap junctions play a role increasingly recognized as important in the cardiovascular system. Cx43 protein abnormalities are associated with a variety of cardiovascular diseases. In addition, several studies have reported that Cx43 regulates various cellular activities. Cx43 is related to arrhythmia, ischemia-reperfusion and heart failure-related apoptosis (Zhang et al., 2017; Ostrakhovitch & Tabibzadeh, 2019; Yang et al., 2019). Previous research by our team showed that APN can protect smooth muscle cell apoptosis induced by CoCl2 by regulating Cx43 (Xiao et al., 2019). However, whether Cx43 modulates the cardioprotective effect of APN is currently unclear.

The phosphatidylinositol-3-kinase (PI3K)/protein kinase B (AKT) pathway is a classical signaling pathway that plays an important role in regulating cell growth, proliferation, autophagy, and apoptosis (Hamzehzadeh et al., 2018 **)**. Various growth factors and cytokines activate the PI3K/AKT signaling pathway, which ultimately phosphorylates AKT. Numerous studies have demonstrated that activated AKT1 has cardioprotective effects (Matsui et al., 2001; Matsui et al., 2002; DeBosch et al., 2006). Several studies have found that AKT2 gene knockout mice have more severe cardiomyocyte apoptosis than normal mice during myocardial ischemia, indicating that AKT2 also has a role in reducing cardiomyocyte apoptosis and protecting the heart (Roberts et al., 2013). A great deal of research has confirmed that the PI3K/AKT signalling pathway can decrease cardiomyocyte apoptosis and protect the heart through a variety of pathways after activation, but many mechanisms remain to be elucidated (Yu et al., 2019). Whether APN protects the myocardium by regulating the PI3K/AKT signaling pathway and whether Cx43 modulates the PI3K/AKT signaling pathway are questions worth exploring.

Our study aimed to establish a septic myocardial injury model to study whether APN preconditioning reduces myocardial injury in sepsis, to explore whether APN can protect myocardial injury through the Cx43 and PI3K/AKT signalling pathways and to investigate the relationship between Cx43 and PI3K/AKT signalling.

## Materials and Methods

### Experimental Animals

C57BL/6J (age 6–8 W; weight 25 ± 5 g) mice were purchased from Beijing Weitong Lihua Experimental Animal Technology (Beijing, China; laboratory animal certificate number: SCXK (jing), 2016-0006). The mice were fostered in the Animal Experiment and Breeding Center of Shihezi University. The entire experiment was carried out in compliance with the requirements of the Animal Experiment Ethics Committee of Shihezi University (Animal Use Certificate SCXK (New) 2018-0001). All mice were housed in an environment with good light and a suitable temperature and humidity. After all mice were adaptively fed for 1 week, 40 mice were randomly divided into four groups, with ten in each group: sham operation (sham group), septic myocardial injury (LPS group), APN combined with LPS (LPS + APN group) and APN (APN group). Each mouse was injected intraperitoneally with 10 mg/kg LPS to establish the septic myocardial injury model. In the sham group, the same amount of normal saline was injected intraperitoneally. Mice in the LPS + APN group were injected intraperitoneally with 6 mg/kg APN 12 h before LPS injection, and mice in the APN group were injected with 6 mg/kg APN. After 12 h, the mice were sacrificed and used for experiments.

### Cell Culture

H9c2 cells were purchased from The Shanghai Institute of Biological Sciences, Chinese Academy of Sciences. This cell line is currently used for screening molecular mechanisms *in vitro*. H9c2 cells were cultured in 10% FBS (Gibco, Carlsbad, CA, USA) and DMEM (Gibco, Thermo Fisher Scientific, Inc.). The control group did not undergo any processing. APN (PeproTech, Rocky Hill, NJ, United States) was added at three concentrations (0.5, 1 and 2 μg/ml) for 2 h, and 1 μg/ml LPS (Sigma, USA) was then added for 24 h. In addition, the Cx43 inhibitor Gap26 (APExBIO Technology LLC, 0.5 μmol/L, 30 min) was added to the preconditioned cells before LPS treatment. The cells were also pre-treated with LY294002 at a concentration of 10 μM for 1 h and then treated with 2 μg/ml APN for 2 h before treatment with LPS for 24 h.

### Cell Viability Assay

Cell viability was estimated by a CCK-8 assay (MultiSciences Lianke Biotech Co., Ltd. Hangzhou). H9c2 cells were cultured in 96-well plates with 1 × 10^4^ cells/well, pre-treated with APN (0.5, 1 and 2 μg/ml) for 2 h, and then incubated with LPS at 37°C for 24 h. After 24 h, 10 μl of the CCK-8 solution was added to each well, and the cells were cultured in an incubator at 37°C for 2 h. The absorbance was measured at 450 nm using enzyme labelling (BioTek Instruments, Inc.).

### Flow Cytometry

The Annexin V-fluorescein isothiocyanate/propidium iodide apoptosis kit (MultiSciences Lianke Biotech Co., Ltd. Hangzhou, China) was used to detect apoptosis. First, H9c2 cells were seeded at a quantity of 1 × 10^6^ cells/ml in a six-well plate. After each group was treated according to the experimental protocol, the cells were detached with 0.25% trypsin, gently triturated, and then washed with PBS after centrifugation. Additionally, the H9c2 cells were combined with Annexin V-FITC, PI and 1× buffer, and the solution was fully mixed and incubated in a dark room at 4°C for 30 min. Quantitative analysis of apoptotic cells was performed by flow cytometry (Becton, Dickinson and Company).

### Western Blot

Treated cells and myocardial tissue were lyzed on ice for at least 15 min. Total protein lysates were collected in 1.5 ml EP tubes, and the protein concentration was determined by the BCA method. Samples with equivalent amounts of protein were separated by SDS-PAGE in 5 × loading buffer. The separated proteins were transferred to PVDF membranes (EMD Millipore), which were subsequently blocked at room temperature with 5% skim milk or 5% BSA for 2 h. The proteins were incubated with antibodies specific for the following proteins overnight in a 4°C shaker: Cx43 (1:1000, Abcam), Bax (1:1000, Abcam), β-actin (1:1000, Beijing Fir Jinqiao Biotechnology), GAPDH (1:1000, Abcam), Bcl2 (1:1000, Abcam), caspase-3 (1:1000, Abcam), caspase-9 (1:1000, Abcam), p-PI3K (1:1000, CST), PI3K (1:1000, Proteintech), p-AKT (1:1000, Proteintech), and AKT (1:1000, Bioworld). Then, the samples were incubated at room temperature for 2 h with the corresponding secondary antibody. The membranes were washed with TBST for 5 min three times, incubated with ECL reagent (GE Healthcare Life Sciences, United Kingdom) and developed. Quantity One software (Bio-Rad, Hercules, CA, United States) was used to analyse the collected images.

### Immunofluorescence

H9c2 cells were evenly distributed in a six-well plate with aseptic slides at a density of 3 × 10^5^ cells/ml. After 24 h, the treated cells were removed and discarded from the culture medium, washed with preheated PBS at 37°C 3 times, and then fixed with rewarmed 40 g/L paraformaldehyde for 10 min. The H9c2 cells were washed with PBS and permeabilized with Triton X-100 for 3 min. Then, the cells were washed with PBS 3 times and incubated with 5% BSA (Sigma-Aldrich, United States) at 37 °C for 30 min. The cells were again washed with PBS 3 times, 100 μL of the primary antibody [(Cx43 (1:100, Abcam)] was added, and the cells were placed in a 4°C wet box overnight. The following day, the cells were washed with PBS 3 times, and the corresponding secondary antibody was added to the wells for incubation at 37°C for 2 h. Subsequently, the cells were washed with PBS three times, the nuclei were stained with DAPI (Solarbio Science and Technology Co., Beijing, China) for 15 min, and the cells were again washed with PBS 3 times. A confocal microscope (Zeiss LSM 510 META, Carl Zeiss AG, Germany) was used to acquire images. Image-Pro Plus 6.0 software (Media Cybernetics, Rockville, MD, United States) was used for semiquantitative analysis of protein expression.

### Hematoxylin-Eosin Staining

The myocardial tissue of each group was placed in 10% formalin overnight and was then dehydrated and embedded in paraffin. According to the instruction manual, the sections were immersed in a concentration gradient of xylene, ethanol and hematoxylin and sealed with neutral gum. An optical microscope (BX51; Olympus, 400×) was used to observe the morphology of cardiomyocytes, cardiac matrix and myofilaments.

### Immunohistochemical Staining

Myocardial tissues were baked at 60°C for 2 h. The tissues were dewaxed and dehydrated with xylene and ethanol, washed with water, repaired with sodium citrate, and incubated with 3% hydrogen peroxide to inhibit the activity of endogenous peroxidase. Then, the tissues were incubated with 5% BSA at 37°C for 1 h. Then, 100 μL of the primary antibody [(Cx43 (1:100, Abcam)] was added, and the tissues were placed in a 4°C wet box overnight. The next day, the corresponding secondary antibody was added to the tissues for incubation at 37°C for 1 h. Signals were developed by using diaminobenzidine as a substrate for 2 min. Six samples were randomly selected from each group, and 5 fields of view were randomly selected from each sample. Images were then acquired under an optical microscope (400×).

### Statistical Analysis

The statistical software SPSS20.0 (IBM Corp.) and GraphPad prism 8.0 (GraphPad Software, La Jolla, CA, United States) were used to analyse the results. The values were expressed as the means ± standard errors, and one-way analysis of variance (ANOVA) and the *t*-test were used to analyse the differences among groups. *p* < 0.05 indicates a significant difference.

## Results

### APN Attenuated the Cytotoxicity Induced by LPS and Reversed LPS-Induced Apoptosis in H9c2 Rat Cardiomyocytes

**FIGURE 1:**
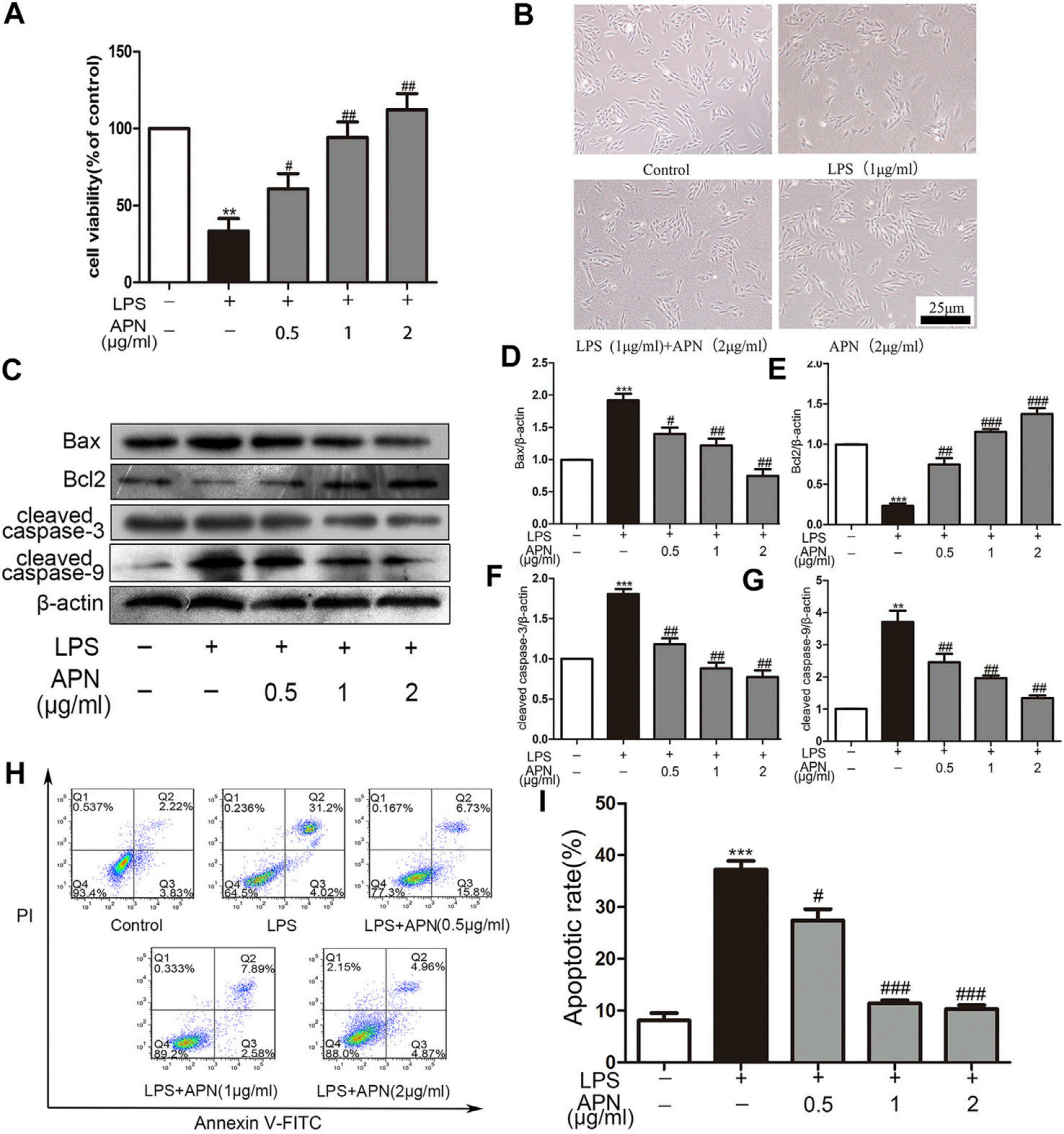
The effects of APN on LPS-induced H9c2 cytotoxicity; cardiomyocyte apoptosis; and Bcl2, Bax, cleaved caspase-3 and cleaved caspase-9 expression in H9c2 cells. **(A)** APN can reduce LPS-induced cytotoxicity. The cells were pre-treated with three concentrations of APN (0.5, 1 and 2 μg/ml) for two hours to assess the preservation function of APN on H9c2 cytotoxicity induced by LPS. **(B)** Cell morphology was observed under an inverted microscope (magnification, ×40). The CCK-8 assay was used to determine cell viability. **(C)** LPS downregulated Bcl2 and upregulated Bax, cleaved caspase-3 and cleaved caspase-9 in H9c2 cells, but these effects were attenuated by APN. **(D–G)** Quantification of Bax, Bcl2, cleaved caspase-3 and cleaved caspase-9 expression. **(H)** The apoptosis rate after treatment with APN and LPS. **(I)** Statistical analysis of the apoptosis rate (*p < 0.05, **p < 0.01, ***p < 0.001 vs. control; ^#^ *p* < 0.05, ^##^ *p* < 0.01, ^###^ *p* < 0.001 vs. the LPS group. The data are shown as the means ± SEs (n = 6)).

The H9c2 cells were pre-treated with three concentrations of APN (0.5, 1 and 2 μg/ml) for 2 h and were then treated with LPS to observe the effect of APN on cardiomyocyte toxicity induced by LPS. The Figure 1A shows that 1 and 2 μg/ml APN had clear protective effects. However, 2 μg/ml APN alone did not affect cell viability (Supplementary the Supplementary Figure S1). In subsequent experiments, 1 μg/ml LPS for 24 h and 2 μg/ml APN for 2 h were used to study the signaling pathways involved in the protection of cardiomyocytes from LPS-induced injury. Cellular morphology was observed by microscopy. As shown in the Figure 1B, cells exposed to LPS for 24 h exhibited disordered cell alignment and extensive cell shrinkage; however, the state of the cells in the co-treatment group was significantly improved. These results suggest that APN attenuates the cytotoxicity of H9c2 cells induced by LPS. Compared with the control group, The LPS downregulated Bcl2 expression and upregulated cleaved caspase-3, Bax, and cleaved caspase-9 expression. The APN reversed these effects in a concentration-dependent manner (the Figures 1C–G). We then performed apoptosis analysis with Annexin V/PI to determine the apoptosis rate. The Q2 region shows late apoptosis, while the Q3 region shows early apoptosis. As presented in the Figures 1H,I, exposure to 1 μg/ml LPS significantly increased the apoptosis rate of H9c2 cells, which was reduced by APN pre-treatment.

### APN Downregulated Cx43 Expression and Activated the PI3K/AKT Signaling Pathway in H9c2 Rat Cardiomyocytes

**FIGURE 2:**
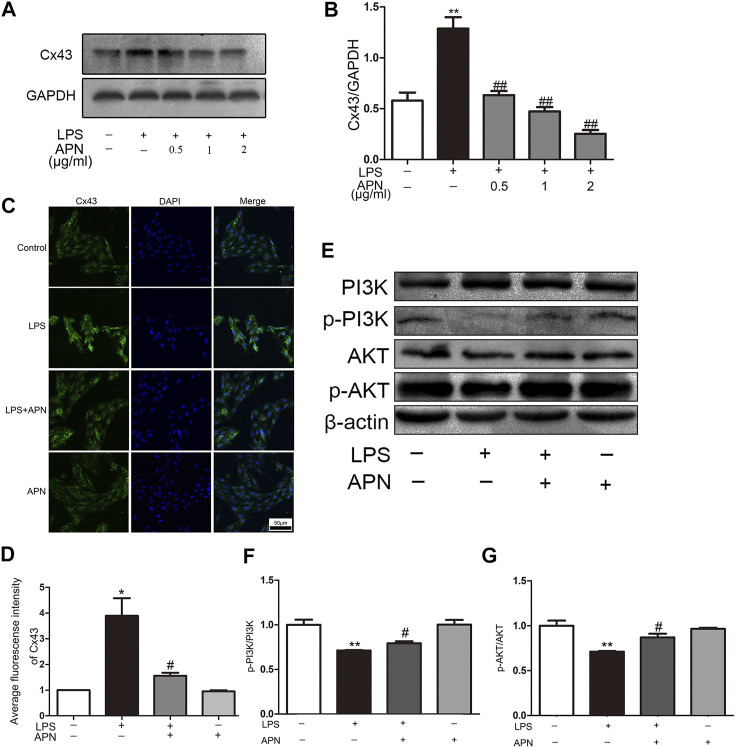
APN downregulates Cx43 expression and activates the PI3K/AKT signaling pathway. **(A)** The effects of LPS and APN on Cx43. **(B)** Statistical analysis of Cx43. **(C)** Fluorescence intensity of Cx43 after treatment with LPS and APN. Scale bar: 50 μm **(D)** Quantitative analysis of the relative fluorescence intensity of Cx43. **(E)** The effects of LPS and APN on PI3K, p-PI3K, AKT, and p-AKT proteins. **(F–G)** Quantification of p-PI3K/PI3K and p-AKT/AKT levels (*P< 0.05, **P< 0.01 vs. control; ^#^ *p* < 0.05, ^##^ *p* < 0.01 vs. the LPS group. The data are shown as the means ± SEs (n = 6)).

The Figures 2A,B shows that LPS upregulated the expression of Cx43, while APN reversed these effects. However, the group treated with APN alone showed no difference in Cx43 expression (Supplementary the Supplementary Figure S2). The immunofluorescence results in the Figures 2C,D show that Cx43 was distributed in the cytoplasm and nucleus. The fluorescence intensity of Cx43 in the LPS group was higher than that in the APN pre-treatment group. This result was consistent with the Western blot analysis results. The Figures 2E–G shows that the p-PI3K and p-AKT protein levels were increased after treatment with 2 μg/ml APN. We previously showed that the PI3K/AKT pathway and Cx43 participate in the protective effect of APN against LPS-induced apoptosis in H9c2 cells.

### Gap26 Reversed LPS-Induced Apoptosis and Activated the PI3K/AKT Signaling Pathway in H9c2 Rat Cardiomyocytes

**FIGURE 3:**
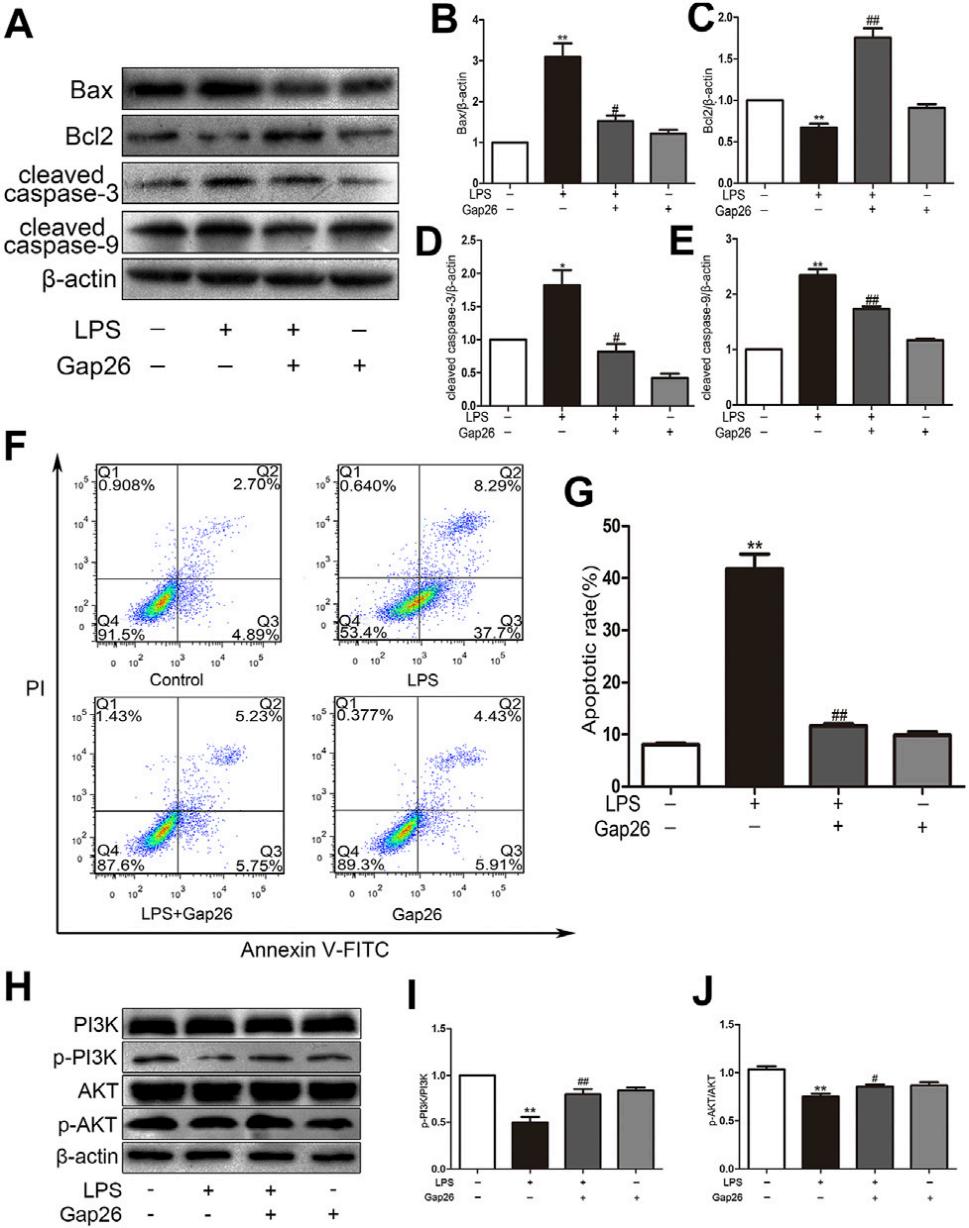
Gap26 reverses LPS-induced apoptosis and activates the PI3K/AKT signalling pathway. **(A)** The effects of LPS and Gap26 on Bax, Bcl2, cleaved caspase-3 and cleaved caspase-9 protein levels. **(B–E)** Statistical analysis. **(F)** The rate of apoptosis after treatment with Gap26 and LPS. **(G)** Statistical analysis. **(H)** The effects of LPS and Gap26 on PI3K, p-PI3K, AKT, and p-AKT proteins. **(I–J)** Statistical analysis (*p < 0.05, **p < 0.01 vs. control, ^#^ *p* < 0.05, ^##^ *p* < 0.01 vs. the LPS group. The data are shown as the means ± SEs (n = 6)).

The H9c2 cells were pre-treated with the Cx43 inhibitor Gap26 at 37°C for 30 min prior to 1 μg/ml LPS treatment for 24 h. The LPS downregulated Bcl2 and upregulated Bax, cleaved caspase-3 and cleaved caspase-9; however, the Gap26 reversed these effects (the Figures 3A–E). The flow cytometry results demonstrated that pre-treatment with Gap26 (0.5 μmol/L for 30 min) significantly changed the percentage of apoptotic H9c2 cells treated with LPS (the Figures 3F,G). The Gap26 alone did not reduce the number of apoptotic cells. Moreover, the Western blot analysis results showed that the p-PI3K and *p*-AKT protein levels were inhibited by LPS, these effects were attenuated by Gap26 (the Figures 3H–J).

### PI3K/AKT Pathway Inhibition Altered the Effect of APN on LPS-Induced Apoptosis in H9c2 Rat Cardiomyocytes

**FIGURE 4:**
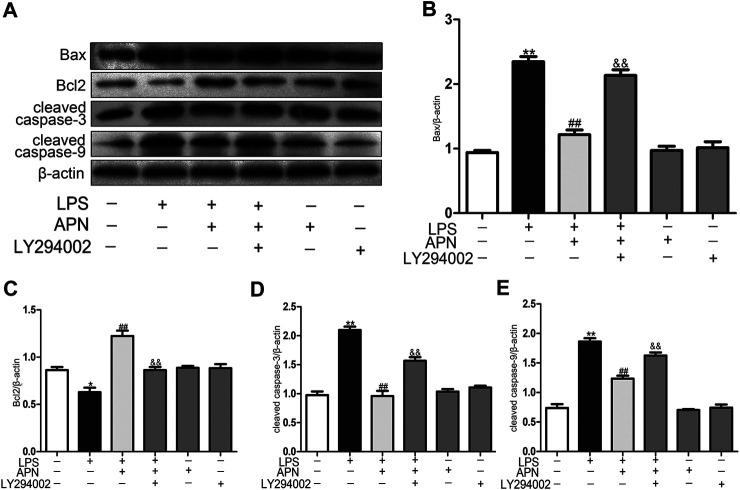
PI3K/AKT pathway inhibition alters the effect of APN on LPS-induced apoptosis in H9c2 cells. **(A)** The effects of APN, LY294002 and LPS on Bax, Bcl2, cleaved caspase-3 and cleaved caspase-9 protein levels. **(B–E)** Statistical analysis. (*p < 0.05, **p < 0.01 vs. control; ^#^ *p* < 0.05, ^##^ *p* < 0.01 vs. the LPS group; ^&^ *p* < 0.05, ^&&^ *p* < 0.01 vs. the LPS + APN group. The data are shown as the means ± SEs (n = 6)).

We sought to determine whether LY294002 reverses the protective effect of APN against LPS-induced apoptosis. Pre-treatment with 10 μM LY294002 for 1 h significantly reversed the protective effect of APN, as demonstrated by the downregulation of Bax, cleaved caspase-3 and cleaved caspase-9 and the upregulation of Bcl2 protein expression (the Figures 4A–E). In conclusion, the PI3K/AKT pathway can participate in the protective function of APN against H9c2 cell apoptosis.

### APN Attenuated Sepsis Myocardial Injury and Downregulated Cx43 Expression in Septic Mice

**FIGURE 5:**
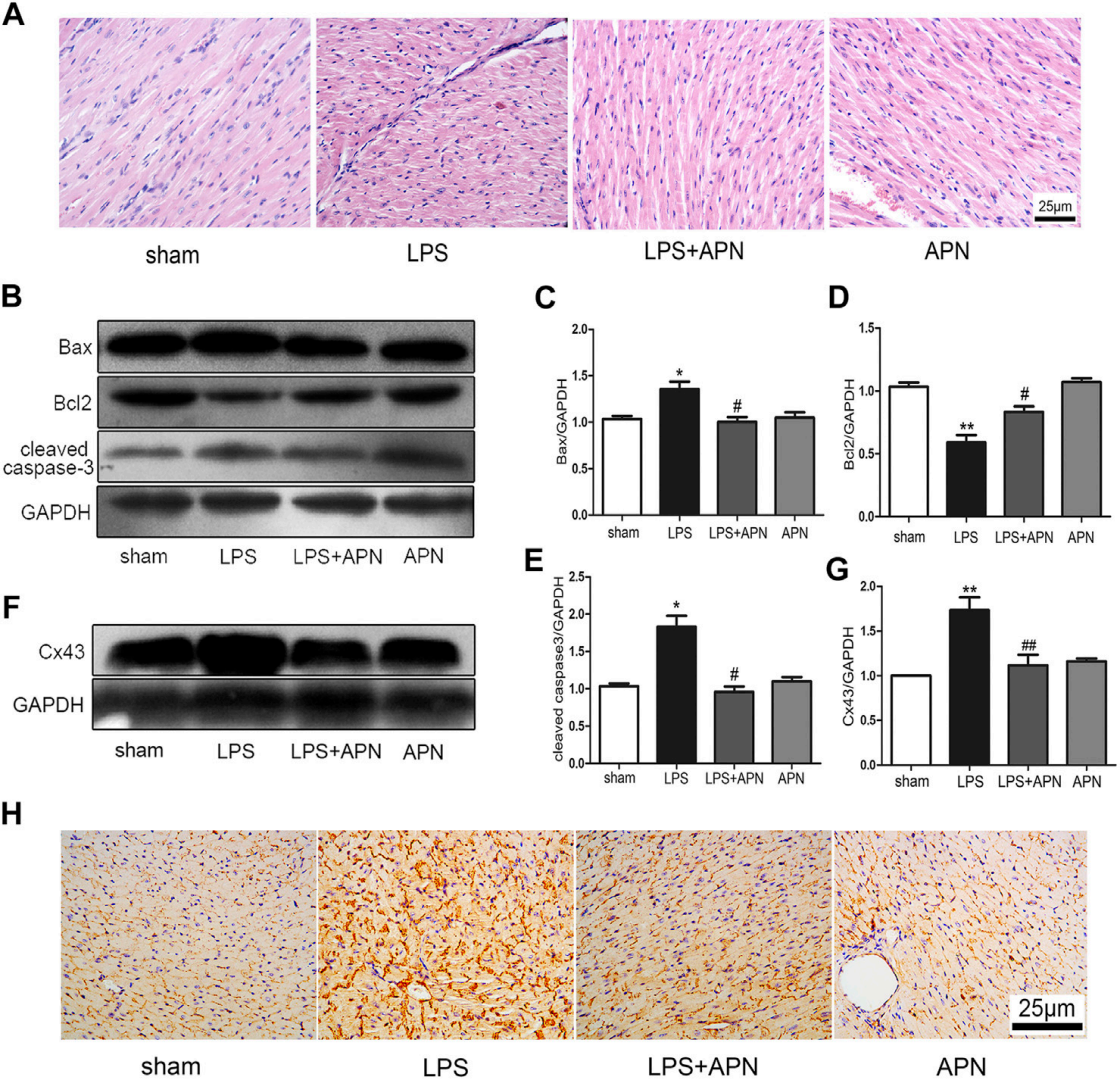
The effects of APN on sepsis-induced myocardial injury. **(A)** HE staining of myocardial tissue at 400×. **(B)** Bcl2 was downregulated and Bax and cleaved caspase-3 were upregulated in myocardial tissues of septic mice, but these effects were attenuated by APN. **(C–E)** Quantification of Bax, Bcl2, and cleaved caspase-3 expression. **(F)** Cx43 was upregulated in myocardial tissue of septic mice, and this effect was changed by APN. **(G)** Quantification of Cx43 expression. **(H)** Immunohistochemical staining showed the expression of Cx43 in myocardial tissue. Scale bar: 25 μm. The brown immunostaining represents Cx43. (*p < 0.05, **p < 0.01 vs. control; ^#^ *p* < 0.05, ^##^ *p* < 0.01 vs. the LPS group. The data are shown as the means ± SEs (n = 6)).

We explored whether APN can protect against myocardial damage in septic mice. The haematoxylin and eosin staining was performed on myocardial tissue to observe differences in the four groups of myocardial tissues. The myocardial fibres were arranged neatly, the cytoplasm was rich and uniform, the intercellular space was normal, the boundary was clear, and there were no pathological changes in the sham group. In the LPS group, inflammatory cell infiltration was observed, accompanied by myocardial cell necrosis. In the LPS + APN group, myocardial cell necrosis was ameliorated, the number of myocardial fibres was increased, and myocardial damage and inflammatory cell infiltration were reduced. In addition, there were no significant changes in the APN group (the Figure 5A). In addition, Bax and cleaved caspase-3 were upregulated in the LPS group, and Bcl2 was downregulated in the LPS group; however, APN reversed these effects (the Figures 5B–E). Next, we further explored whether Cx43 is involved in the protective role of APN against myocardial damage in septic mice. Cx43 was upregulated in septic mice, while APN downregulated the expression of Cx43 (the Figures 5F–H).

## Discussion

Overall, our study indicates that APN protects against LPS-induced cardiomyocyte apoptosis. In addition, the data showed that APN activates the PI3K/AKT signaling pathway and downregulates the expression of Cx43. PI3K/AKT pathway inhibition altered the inhibitory effect of APN on LPS-induced apoptosis in H9c2 cells. The Cx43 selective inhibitor Gap26 reversed LPS-induced apoptosis and activated PI3K/AKT signaling pathway inhibited by LPS. In addition, in septic mice, APN protected the myocardium against sepsis-related damage by upregulating Cx43. Therefore, this study provides proof of concept that APN attenuates LPS-induced apoptosis by regulating the Cx43/PI3K/AKT pathway.

Sepsis has a rapid onset, abrupt development, and a high mortality rate. Accumulating evidence has shown that most patients with septic shock suffer from myocardial depression (Xu et al., 2020). LPS treatment mimics the pathological characteristics of myocardial infarction patients, such as myocardial morphology and function (Sánchez-Villamil et al., 2016; Shrestha et al., 2017). LPS is often used to induce myocardial injury. Some research has suggested that LPS can activate the apoptotic signaling pathway in cardiomyocytes (Wu et al., 2017; Xu et al., 2018; Zhang et al., 2020). Our study used an LPS-induced endotoxaemic heart injury model in H9c2 rat cardiomyocytes and C57BL/6J mice. Tan et al. found that LPS induced AC16 cell apoptosis (Tan et al., 2019). Herein, we showed that in H9c2 cells LPS (1 μg/ml) reduced cell viability; upregulated the levels of Bax, cleaved caspase-3 and cleaved caspase-9; and downregulated Bcl2 expression. In addition, the data showed that Bax and cleaved caspase-3 were upregulated and Bcl2 downregulated in septic mice.

APN, an endogenous bioactive polypeptide or protein secreted by adipocytes, is a high molecular weight (HMW) APN. Previous studies have shown that APN may have cardioprotective effects (Wang H. et al., 2020). In this study, pre-treatment with three concentrations of APN (0.5, 1, and 2 μg/ml) reversed the decrease in cell viability caused by LPS. Our results therefore show that APN protects against cardiotoxicity caused by LPS. Xiao et al. reported that APN reversed CoCl2-induced apoptosis in SMCs (Xiao et al., 2019). Thus, our results are consistent with those previously reported. We also found that APN can protect against myocardial injury in septic mice.

Next, we explored the potential mechanism by which APN protects against myocardial injury induced by LPS. Apoptotic proteins are downstream effectors of Akt. Recently, data have shown that PI3K/AKT pathway activation inhibits cardiomyocyte apoptosis. Furthermore, PI3K/AKT signaling pathway activation may delay the progression of heart failure (Takatani et al., 2004). Shang X et al. found that resveratrol activated the PI3K/AKT signalling pathway and inhibited the NF-κB signaling pathway and related inflammatory factors in myocardial injury in septic rats (Shang et al., 2019). Chang JH et al. reported that PI3K/AKT signaling pathway activation protected against cardiomyocyte injury during ischemia-reperfusion in diabetic rats (Chang et al., 2020). We found that APN increased the levels of phosphorylated PI3K and AKT. Moreover, APN reversed the decrease in PI3K and AKT phosphorylation induced by LPS, which indicates that APN can activate the PI3K/AKT pathway in H9c2 cells. These findings are in accordance with the previous observation that APN upregulated the PI3K/AKT pathway in a myocardial post-ischaemic injury model (Wang et al., 2013). In addition, we used LY294002 to evaluate the effect of the PI3K/AKT pathway on the function of APN against LPS-induced apoptosis in H9c2 rat cardiomyocytes. The results showed that LY294002 reversed the protective effects of APN on LPS-induced apoptosis in H9c2 cells, as demonstrated by upregulation of Bax, cleaved caspase-3, and cleaved caspase-9 and downregulation of Bcl2. Wei et al. found that APN protects H9c2 cells against palmitic acid-induced apoptosis through the Akt signalling pathway (Dong et al., 2012). These data are consistent with our findings. Here, we examined whether the PI3K/AKT signaling pathway plays an important role in the APN-mediated protection of H9c2 cells.

Gap junctions are membrane channel structures that exist between adjacent cells, mediate the transmission of information between cells and have important biological functions (Pieperhoff and Franke, 2007). Cx43 is a major gap junction protein in mammalian ventricular myocytes. Allen et al. (Allen, 1992) found that in various heart diseases such as hypertrophic cardiomyopathy, heart failure and ischemic cardiomyopathy, the distribution and expression of Cx43 are altered. Cx43 plays a significant role in cell growth, proliferation and apoptosis. Recently, a study showed that Cx43 gene knockout or accelerated degradation protected astrocytes from apoptosis under ischemic stress (Wang X. et al., 2020). Ma JW et al. found that Cx43 inhibition attenuated oxidative stress and apoptosis in HUVECs (Ma et al., 2020). In our study, inhibiting Cx43 attenuated LPS-induced apoptosis. After treatment with LPS, the expression of Cx43 was reduced, and apoptosis gradually increased in H9c2 cells and septic mice. Different concentrations of APN downregulated Cx43 expression. Therefore, Cx43 also plays a key role in APN-mediated protection against myocardial injury in sepsis. Moreover, we found that Gap26 activated the PI3K/AKT signaling pathway. A recent study showed that disruption of Cx43 also enhanced the phosphorylation of Akt (Wang Y. et al., 2020). This finding is consistent with our results. Our study showed that APN can protect against myocardial injury through the Cx43 and PI3K/AKT signaling pathways. Interestingly, a study found that treatment with a PI3K inhibitor (LY294002) reduced the expression of Cx43 in diabetes models (Bi et al., 2017). Therefore, our follow-up experiments will continue to explore whether changes in the PI3K/AKT signaling pathway related to APN-mediated protection against myocardial injury cause changes in Cx43 expression. In addition, we will continue to explore Cx43 phosphorylation in future research.

## Conclusion

Our study demonstrates that APN protects against LPS-induced injury by regulating Cx43 expression and activating the PI3K/AKT signaling pathway. These results suggest that APN may be an effective treatment for cardiovascular disease.

## Data Availability

The original contributions presented in the study are included in the article/Supplementary Material, further inquiries can be directed to the corresponding authors.

## References

[B1] Allen A. (1992). The Cardiotoxicity of Chemotherapeutic Drugs. Semin. Oncol. 19 (5), 529–542. 1411651

[B2] Alvarez S. Vico T. Vanasco V. (2016). Cardiac Dysfunction, Mitochondrial Architecture, Energy Production, and Inflammatory Pathways: Interrelated Aspects in Endotoxemia and Sepsis. Int. J. Biochem. Cel Biol. 81, 307–314. 10.1016/j.biocel.2016.07.032 27477311

[B3] Bi Y. Wang G. Liu X. Wei M. Zhang Q. (2017). Low-after-high Glucose Down-Regulated Cx43 in H9c2 Cells by Autophagy Activation via Cross-Regulation by the PI3K/Akt/mTOR and MEK/ERK1/2 Signal Pathways. Endocrine 56 (2), 336–345. 10.1007/s12020-017-1251-3 28181145

[B4] Blanco J. Muriel-Bombín A. Sagredo A. V. Taboada F. Gandía F. Tamayo L. (2008). Incidence, Organ Dysfunction and Mortality in Severe Sepsis: a Spanish Multicentre Study. Crit. Care 12, R158. 10.1186/cc7157 19091069 PMC2646323

[B5] DeBosch B. Sambandam N. Weinheimer C. Courtois M. Muslin A. J. (2006). Akt2 Regulates Cardiac Metabolism and Cardiomyocyte Survival. J. Biol. Chem. 281, 32841–32851. 10.1074/jbc.M513087200 16950770 PMC2724003

[B6] Chang J.-H. Jin M.-M. Liu J.-T. (2020). Dexmedetomidine Pretreatment Protects the Heart against Apoptosis in Ischemia/reperfusion Injury in Diabetic Rats by Activating PI3K/Akt Signaling In Vivo and In Vitro. Biomed. Pharmacother. 127, 110188. 10.1016/j.biopha.2020.110188 32407987

[B7] Dong W.C. Yan L. Yun Z. H. (2012). Globular Adiponectin Protects H9c2 Cells from Palmitate-Induced Apoptosis via Akt and ERK1/2 Signaling Pathways. Lipids Health Dis. 11, 135. 10.1186/1476-511X-11-135 23050488 PMC3540014

[B9] Essick E. E. Ouchi N. Wilson R. M. Ohashi K. Ghobrial J. Shibata R. (2011). Adiponectin Mediates Cardioprotection in Oxidative Stress-Induced Cardiac Myocyte Remodeling. Am. J. Physiology-Heart Circulatory Physiol. 301, H984–H993. 10.1152/ajpheart.00428.2011 PMC3191107 21666115

[B8] Essick Eric E. Wilson Richard M. Pimentel David R. (2013). Adiponectin Modulates Oxidative Stress-Induced Autophagy in Cardiomyocytes. [J] .PLoS One 8, e68697. 10.1371/journal.pone.0068697 PMC3716763 23894332

[B10] Fallach R. Shainberg A. Avlas O. Fainblut M. Chepurko Y. Porat E. (2010). Cardiomyocyte Toll-like Receptor 4 Is Involved in Heart Dysfunction Following Septic Shock or Myocardial Ischemia. J. Mol. Cell Cardiol. 48, 1236–1244. 10.1016/j.yjmcc.2010.02.020 20211628

[B11] Ghantous C. M. Azrak Z. Hanache S. (2015). Differential Role of Leptin and Adiponectin in Cardiovascular System. [J]. Int. J. Endocrinol. 2015, 534320. 10.1155/2015/534320 PMC4433709 26064110

[B12] Hamzehzadeh L. Atkin S. L. Majeed M. Butler A. E. Sahebkar A. (2018). The Versatile Role of Curcumin in Cancer Prevention and Treatment: a Focus on PI3K/AKT Pathway. J. Cel. Physiol. 233, 6530–6537. 10.1002/jcp.26620 29693253

[B13] Han Fang. Judd Robert L. (2018). Adiponectin Regulation and Function. Compr. Physiol. 8, 1031–1063. 10.1002/cphy.c170046 29978896

[B14] Luo M. Yan D. Sun Q. Tao J. Xu L. Sun H. (2020). Ginsenoside Rg1 Attenuates Cardiomyocyte Apoptosis and Inflammation via the TLR4/NF‐kB/NLRP3 Pathway. J. Cel Biochem 121, 2994–3004. 10.1002/jcb.29556 31709615

[B15] Ma J. W. Ji D. D. Li Q. Q. Zhang T. Luo L. (2020). Inhibition of Connexin 43 Attenuates Oxidative Stress and Apoptosis in Human Umbilical Vein Endothelial Cells[J]. BMC Pulm. Med. 20 (1). 10.1186/s12890-019-1036-y PMC6975083 31964358

[B17] Matsui T. Li L. Wu J. C. Cook S. A. Nagoshi T. Picard M. H. (2002). Phenotypic Spectrum Caused by Transgenic Overexpression of Activated Akt in the Heart. J. Biol. Chem. 277, 22896–22901. 10.1074/jbc.m200347200 11943770

[B16] Matsui T. Tao J. del Monte F. Lee K.-H. Li L. Picard M. (2001). Akt Activation Preserves Cardiac Function and Prevents Injury after Transient Cardiac Ischemia In Vivo. Circulation 104, 330–335. 10.1161/01.cir.104.3.330 11457753

[B18] Merx M. W. Weber C. (2007). Sepsis and the Heart. Circulation 116, 793–802. 10.1161/circulationaha.106.678359 17698745

[B19] Ostrakhovitch E. A. Tabibzadeh S. (2019). Homocysteine and Age-Associated Disorders. Ageing Res. Rev. 49, 144–164. 10.1016/j.arr.2018.10.010 30391754

[B20] Pieperhoff S. Franke W. W. (2007). The Area Composita of Adhering Junctions Connecting Heart Muscle Cells of Vertebrates - IV: Coalescence and Amalgamation of Desmosomal and Adhaerens Junction Components - Late Processes in Mammalian Heart Development. Eur. J. Cel Biol. 86 (7), 377–391. 10.1016/j.ejcb.2007.04.001 17532539

[B21] Poveda-Jaramillo R. (2021). Heart Dysfunction in Sepsis. J. Cardiothorac. Vasc. Anesth. 35, 298–309. 10.1053/j.jvca.2020.07.026 32807603

[B22] Roberts D. J. Tan-Sah V. P. Smith J. M. Miyamoto S. (2013). Akt Phosphorylates HK-II at Thr-473 and Increases Mitochondrial HK-II Association to Protect Cardiomyocytes. J. Biol. Chem. 288, 23798–23806. 10.1074/jbc.m113.482026 23836898 PMC3745326

[B23] Sammon J. D. Klett D. E. Sood A. Olugbade K. Schmid M. Kim S. P. (2015). Sepsis after Major Cancer Surgery. J. Surg. Res. 193, 788–794. 10.1016/j.jss.2014.07.046 25167780

[B24] Sánchez-Villamil J. P. D’Annunzio V. Holod P. S. Rebagliati I. Pérez H. Peralta J. G. (2016). Cardiac-specific Overexpression of Thioredoxin 1 Attenuates Mitochondrial and Myocardial Dysfunction in Septic Mice. Int. J. Biochem. Cel Biol. 81, 323–334. 10.1016/j.biocel.2016.08.045 27592449

[B25] Shibata R. Sato K. Pimentel D. R. Takemura Y. Kihara S. Ohashi K. (2005). Adiponectin Protects against Myocardial Ischemia-Reperfusion Injury through AMPK- and COX-2-dependent Mechanisms. Nat. Med. 11, 1096–1103. 10.1038/nm1295 16155579 PMC2828682

[B26] Shrestha G. S. Kwizera A. Baelani G. J. I. Azevedo L. C. P. Pattnaik R. Haniffa R. (2017). International Surviving Sepsis Campaign Guidelines 2016: the Perspective from Low-Income and Middle-Income Countries. Lancet Infect. Dis. 17, 893–895. 10.1016/s1473-3099(17)30453-x 28845789

[B27] Takatani T. Takahashi K. Uozumi Y. Matsuda T. Ito T. Schaffer S. W. (2004). Taurine Prevents the Ischemia-Induced Apoptosis in Cultured Neonatal Rat Cardiomyocytes through Akt/caspase-9 Pathway. Biochem. Biophysical Res. Commun. 316 (2), 484–489. 10.1016/j.bbrc.2004.02.066 15020243

[B28] Tan J. Sun T. Shen J. Zhu H. Gong Y. Zhu H. (2019). FAM46C Inhibits Lipopolysaccharides-Induced Myocardial Dysfunction via Downregulating Cellular Adhesion Molecules and Inhibiting Apoptosis. Life Sci. 229, 1–12. 10.1016/j.lfs.2019.03.048 30910647

[B30] Wang H. Gao Y.-X. Wu Y.-N. Li C. Duan J. (2020). Association between Plasma Adiponectin Levels and Left Ventricular Systolic Dysfunction in Sepsis Patients. J. Crit. Care 60, 195–201. 10.1016/j.jcrc.2020.06.020 32854089

[B29] Wang T. Mao X. Li H. Qiao S. Xu A. Wang J. (2013). N-acetylcysteine and Allopurinol Up-Regulated the Jak/STAT3 and PI3K/Akt Pathways via Adiponectin and Attenuated Myocardial Postischemic Injury in Diabetes. Free Radic. Biol. Med. 63 (14), 291–303. 10.1016/j.freeradbiomed.2013.05.043 23747931

[B31] Wang X. Feng L. Xin M. Hao Y. Wang X. Shang P. (2020). Mechanisms Underlying Astrocytic Connexin-43 Autophagy Degradation during Cerebral Ischemia Injury and the Effect on Neuroinflammation and Cell Apoptosis. Biomed. Pharmacother. 127, 110125. 10.1016/j.biopha.2020.110125 32361163

[B32] Wang Y. Wang W. Wu X. Li C. Huang Y. Zhou H. (2020). Resveratrol Sensitizes Colorectal *Cancer* Cells to Cetuximab by Connexin 43 Upregulation-Induced Akt Inhibition[J]. Front. Oncol. 10, 383. 10.3389/fonc.2020.00383 32318334 PMC7155766

[B33] Wu B. Ni H. Zhuang J. X. Zhang J. Qi Z. Chen Q. (2017). The Impact of Circulating Mitochondrial DNA on Cardiomyocyte Apoptosis and Myocardial Injury after TLR4 Activation in Experimental Autoimmune Myocarditis. Cell Physiol Biochem 42, 713–728. 10.1159/000477889 28618428

[B34] Xiao J. Yang R. Qin X. Zhang Z. Li X. Li L. (2019). A Role of AMPK and Connexin 43 in the Suppression of CoCl2-Induced Apoptosis of Spiral Modiolar Artery Smooth Muscle Cells by Adiponectin. Life Sci. 238, 116876. 10.1016/j.lfs.2019.116876 31655194

[B35] Shang X. Lin K. Yu R. Zhu P. Zhang Y. Wang L. (2019). Resveratrol Protects the Myocardium in Sepsis by Activating the Phosphatidylinositol 3-Kinases (PI3K)/AKT/Mammalian Target of Rapamycin (mTOR) Pathway and Inhibiting the Nuclear Factor-Κb (NF-Κb) Signaling Pathway. Med. Sci. Monit. 25, 9290–9298. 10.12659/MSM.918369 31806860 PMC6911307

[B36] Xu J. Lin C. Wang T. Zhang P. Liu Z. Lu C. (2018). Ergosterol Attenuates LPS-Induced Myocardial Injury by Modulating Oxidative Stress and Apoptosis in Rats. Cel Physiol Biochem 48, 583–592. 10.1159/000491887 30021198

[B37] Xu Q. Xiong H. Zhu W. Liu Y. Du Y. (2020). Small Molecule Inhibition of Cyclic GMP-AMP Synthase Ameliorates Sepsis-Induced Cardiac Dysfunction in Mice. Life Sci. 260, 118315. 10.1016/j.lfs.2020.118315 32835697

[B38] Yang Y. Yan X. Xue J. Zheng Y. Chen M. Sun Z. (2019). Connexin43 Dephosphorylation at Serine 282 Is Associated with Connexin43-Mediated Cardiomyocyte Apoptosis. Cell Death Differ 26, 1332–1345. 10.1038/s41418-019-0277-x 30770876 PMC6748098

[B39] Yu T. Liu D. Gao M. Yang P. Zhang M. Song F. (2019). Dexmedetomidine Prevents Septic Myocardial Dysfunction in Rats via Activation of α7nAChR and PI3K/Akt- Mediated Autophagy. Biomed. Pharmacother. 120, 109231. 10.1016/j.biopha.2019.109231 31546082

[B41] Zhang T. Liu C.-F. Zhang T.-N. Wen R. Song W.-L. (2020). Overexpression of Peroxisome Proliferator-Activated Receptor γ Coactivator 1-α Protects Cardiomyocytes from Lipopolysaccharide-Induced Mitochondrial Damage and Apoptosis. Inflammation 43, 1806–1820. 10.1007/s10753-020-01255-4 32529514

[B40] Zhang Z. Zhao L. Zhou Y. Lu X. Wang Z. Wang J. (2017). Taurine Ameliorated Homocysteine-Induced H9C2 Cardiomyocyte Apoptosis by Modulating Endoplasmic Reticulum Stress. Apoptosis 22, 647–661. 10.1007/s10495-017-1351-9 28229251

