# Supplementary figures and images for: Adiponectin Attenuates Lipopolysaccharide-induced Apoptosis by Regulating the Cx43/PI3K/AKT Pathway
Source: Front Pharmacol. 2021 May 18;12:644225. doi: 10.3389/fphar.2021.644225 (PMC8167433; doi:10.3389/fphar.2021.644225)

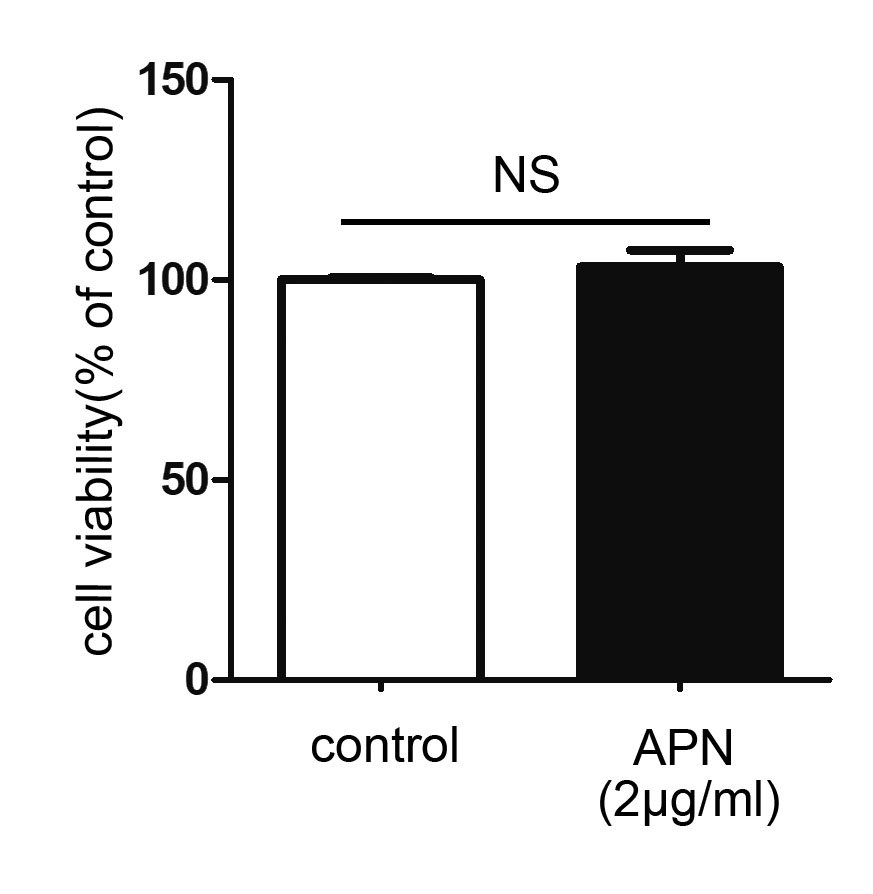

Supplement: Supplementary file 1 [file Image1.JPEG]

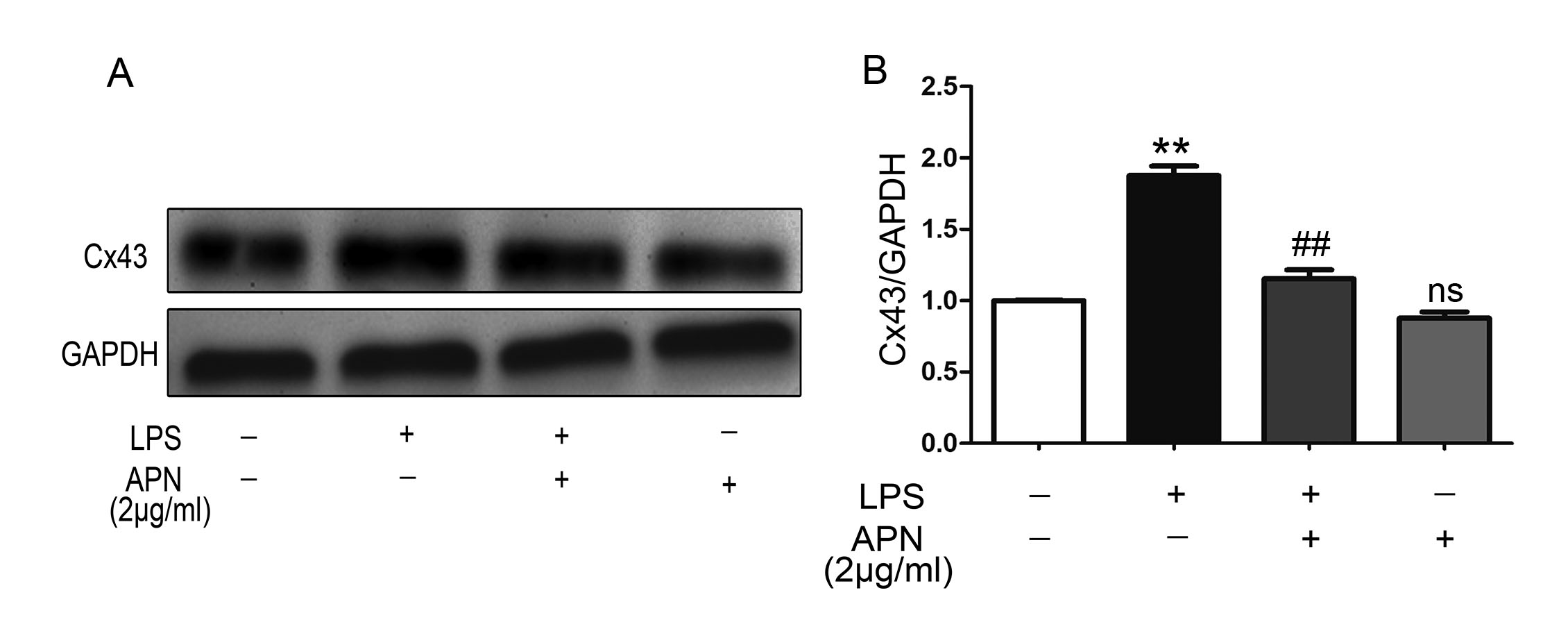

Supplement: Supplementary file 2 [file Image2.JPEG]
